# Age-Related Changes in the Temperature of the Lumbar Spine Measured by Passive Microwave Radiometry (MWR)

**DOI:** 10.3390/diagnostics13213294

**Published:** 2023-10-24

**Authors:** Alexander V. Tarakanov, Alexander A. Tarakanov, Elizaveta G. Skorodumova, Neil Roberts, Toshi Kobayshi, Sergey G. Vesnin, Vladimir Zelman, Igor Goryanin

**Affiliations:** 1 Department of Emergency Medicine, Rostov State Medical University, 344022 Rostov-on-Don, Russia; dr-tarakanov@yandex.ru (A.V.T.); scenar.neuro@gmail.com (A.A.T.); 2 Dzhanelidze Research Institute of Emergency Care, 192242 St. Petersburg, Russia; lisavetta91@mail.ru; 3 The Queen’s Medical Research Institute (QMRI), University of Edinburgh, Edinburgh EH8 9YL, UK; neil.roberts@ed.ac.uk; 4 MMWR Ltd., Edinburgh EH10 5LZ, UK; binsei.nd@gmail.com (T.K.); vesnin47@gmail.com (S.G.V.); 5 Keck School of Medicine, University of South California, Los Angeles, CA 90089, USA; vzelman@usc.edu; 6 Biological Systems Unit, Okinawa Institute Science and Technology, Okinawa 904-0495, Japan; 7 School of Informatics, University of Edinburgh, Edinburgh EH8 9YL, UK

**Keywords:** passive microwave radiometry (MWR), lumbar vertebrae, biological age

## Abstract

A study was conducted to determine the age dependence of the temperature of the low back in the region of the five lumbar vertebrae by using passive microwave radiometry (MWR). The rationale for the study is that the infrared brightness on which the temperature measurement is based will be dependent upon blood circulation and thus on metabolic, vascular, and other regulatory factors. The brightness and infrared temperatures were determined in five zones above each of the medial, left, and right lateral projections of the vertebrae. A total of 115 healthy subjects were recruited, aged between 18 and 84 years. No significant differences in infrared temperature were detected. As predicted, brightness temperature increased until 25 years old and then gradually decreased. In subjects over 70 years of age, compared with those aged 60–70 years, there is a significant increase in brightness temperature at the level of 3–5 lumbar vertebrae by 0.3–0.7 °C. This is interpreted as indicating that individuals who have lived to an advanced age successfully maintain metabolic and regenerative processes. The benchmark data that has been obtained can be usefully employed in future studies of the aetiology of low back pain. In particular, the prospect exists for the technology to be used to provide a non-invasive biomarker to evaluate the effectiveness of antiaging therapies.

## 1. Introduction

Lower back pain (LBP) is the most common musculoskeletal disorder. This is the cost of walking upright, which is exacerbated by factors such as genetic predisposition, working conditions/environment, increasing age, and various diseases [1]. According to literature data, up to 50–99% of the population experienced a feeling of discomfort or an episode of back pain at least once in a lifetime. There is a big gap between basic science research that tries to identify the underlying biological causes of a problem and applied rehabilitation research. Such research pragmatically focuses on modifiable factors influencing back pain outcomes. At the same time, during the internal structure of back pain, in up to 60–80% of cases, the lumbar-sacral spine fails due to its biomechanics and an increased load on this part of the spine [2,3]. Age and pathological degenerative changes affect both the bone and soft tissue structures of the spine. All this leads to changes in the vertebral motor segments. At the same time, it is generally accepted that age-related processes are normal anatomical changes [4,5]. Changes in the lower back are accompanied by changes in metabolism and natural blood circulation in bones, joints, ligaments, and muscle apparatus. As a result, it follows that there will be changes in the temperature of the tissues of the vertebral segment. Temperature is the main property of any living system. Even slight fluctuations have a profound effect on the body. Changes in core body temperature at 1 °C could lead to significant changes in organisms, the cardiovascular system, etc. Temperature maintenance is determined by the level and demands of metabolism, the state of the vascular channels, and the effectiveness of regulation. Naturally, with age, body systems will change, and therefore temperature can serve as an indicator of age. Within the concept of predictors of biological age, a biomarker can be defined as an indicator correlated with chronological age, which brings additional information to risk assessments of age-related conditions on top of chronological age [6]. In this vein, temperature changes in the lumbar spine may become one of the predictors of local ageing and determine the biological age of the spine, which was the goal of our research [7]. MRI and fMRI are interesting technologies, but they are not always available in medical centres for long-term temperature monitoring, and the cost of such equipment is quite high [8,9,10].

The zero-heat-flux method (ZHFM) uses a temperature sensor and an electric heater to create an isothermal tunnel where the infrared surface is heated to the temperature of the brain when the sensor is placed on the forehead. This is a promising method based on temperature measurement using four thermistors. It has low power consumption and good prospects for miniaturization. Conducted clinical trials have shown that the results of temperature measurement using ZHFM DHFM correlate well with the results obtained by other methods.

It should be borne in mind that these methods allow monitoring of the temperature, for example, of the brain at one point and do not provide information about the temperature distribution in different areas of the body. Moreover, to achieve the thermal equilibrium of the sensor, a rather long time is required (20–30 min) and, in some cases, up to 60 min [11,12,13]. The dual-heat-flux method (DHFM) is a relatively new method that calculates the brightness temperature based on the heat flux inside a probe. Unlike ZHFM, this method does not require a heater but uses four temperature sensors located at different distances from the head surface [14]. Microwave radiometry (MWR) [15] allows non-invasive measuring of the temperature at one point in 5–7 s. It is possible to measure the internal temperature in several zones and build an internal temperature field. The full cycle of measurements required to build the field of internal temperatures in certain areas of the spine is about 5–7 min. The MWR is based on measuring the intensity of the intrinsic electromagnetic radiation of the patient’s internal tissues in the microwave frequency range. The intensity of this radiation is directly proportional to the temperature of the tissues. This property of heated bodies is used to measure the average temperature. The method of passive microwave radiometry (MWR) is fundamentally new in biomedical research. Previously, measurements of the area of the lumbar vertebrae were performed using infrared (IR) thermography. However, using IR thermography makes it possible to measure and visualise the temperature of the infrared only, and microwave radiometry provides information about the temperature at a depth of several centimeters. fMRI could be used to measure brightness temperature, but it is very expensive for studies on healthy volunteers. The following methods of non-invasive measurement of brightness temperature are most widely used today.

## 2. Materials and Methods

The studies were carried out in the “Problem Scientific Laboratory of Physical Methods Diagnostics and Treatment” at Rostov State Medical University (Rostov State Medical University) and the rehabilitation centre “Mir” (Khutor Krasny Desant). Informed consent was obtained from volunteers; the positive decision of the independent ethical committee No. 10/19 dated 30 May 2019, RostGMU. Inclusion criteria: healthy volunteers without acute, subacute, or chronic low back pain in the past 6 months. Criteria for non-inclusion: manifestations of radiculopathy and myelopathy, congenital anomalies of the spine, ankylosing spondylitis, reactive arthritis, rheumatoid arthritis, and patients with neuropathic pain. Before the study, when interviewing volunteers for possible early causes of back pain (red flags), they were recommended to consult medical specialists and undergo medical examinations [7]. MWR data were obtained from 115 healthy volunteers (18–84 years old). We formed 7 groups with an age resolution of 10 years: 1 gr.—18–20 years old, *n* = 15; 2 gr. 21–30 years old, *n* = 18; 3 gr.—31–40 years old, *n* = 12; 4 gr.—41–50 years old, *n* = 6; 5 gr.—51–60 years old, *n* = 19; 6 gr.—61–69 years old, *n* = 22 (men—7, women—15); 7 gr.—71–84 years old, *n* = 23 (10 men, 13 women). The gender distribution in the other groups was approximately equal in the ratio of men to women. In clinical practice, the body mass index, which we determined in volunteers, is most often used to assess body weight. If they had insufficient (deficit) body weight or grade 1 obesity, they were excluded from the study. Body mass index (BMI) ranged from 18.5–30 kg/m^2^.

The temperature measured with a radiometer is called the brightness temperature and is the temperature in the volume (cylinder) directly under the antenna (3–7 cm, depending on the diameter of the antenna and type of tissue). The principles and clinical data are described in detail [15,16,17,18]. Temperature measurements in the region of the lumbar vertebrae were obtained using the MWR2020 system (former RTM-01-RES) (MMWR Ltd., Edinburgh, UK). The system is approved for clinical use in FSU countries and has a CE class I. Operates in the frequency range from 1 to 4 GHz; antenna applicator diameter is 39 mm; claimed accuracy is ±0.2 °C for both internal and infrared temperatures [19].

The measurement of internal tissue temperature depends on various factors, such as the moisture content of the tissues and the frequency range used for measurement. The measurement depth of tissues changes with an increase in the frequency range. For instance, at a frequency of 1 GHz, the measurement depth for muscles is 40 mm, and for fat, it is 232 mm. However, as the frequency increases, the measurement depth decreases. For most tissues, at frequencies above 4.5 GHz, the measurement depth becomes less than 1 cm. Therefore, non-invasive tissue temperature measurement is generally carried out in the frequency range of 1 GHz to 4 GHz. Although the measurement depth is significantly higher at a frequency of 1 GHz compared to 3.8 GHz, using a frequency range of 1–2.4 GHz is not recommended due to the high level of spurious interference from mobile phones and other wireless devices. This interference can be reduced by performing the self-radiation measurement in shielded rooms. An interference-proof radiometer such as the MWR2020 operating in the range of 3.4–4.2 GHz can be used to measure brightness temperature without requiring special room shielding. MMWR2020 can provide additional information about brightness and infrared temperature simultaneously.

**Figure 1:**
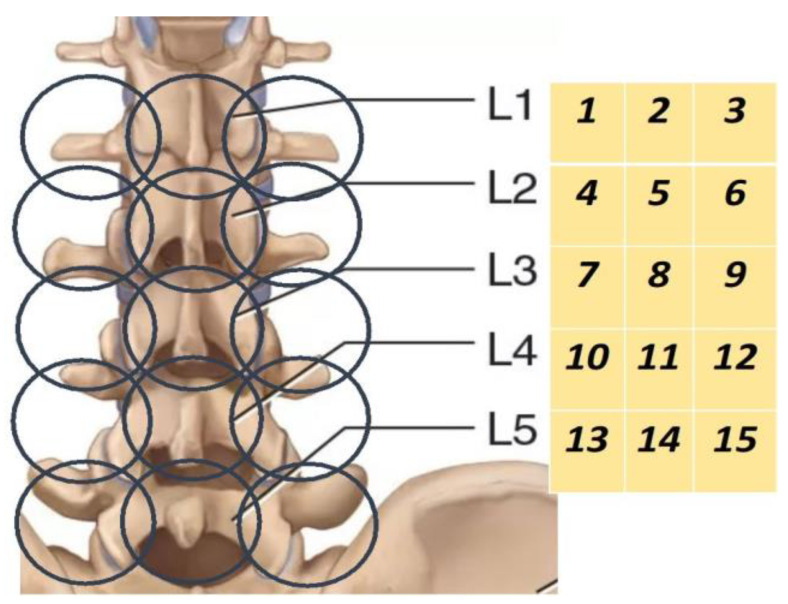
Examination points. MWR analysis was carried out in measurement zones 2, 5, 8, 11, and 14.

Brightness and infrared temperatures were assessed over 15 measurement zones (Figure 1), with t min and t max also taken at each measurement zone. Thermal asymmetry was visually represented by temperature fields. In this paper, we present only an analysis of the brightness temperature above the spinous processes of the lumbar vertebrae. The choice of the projection of the spinous processes is associated with their high verification during measurement.

Each temperature value was visualized, and the same temperature is connected by isotherms. There are transitional colours between the coldest (blue) and hottest (red) zones. They allow for visualising temperature transitions. Since there was no normal distribution of quantitative data, the results were described using the median (Me) and the lower and upper quartiles (Q1–Q3). A comparison of two groups according to a quantitative indicator whose distribution is different from normal was performed using the Kruskal-Wallis test with the Dwass-Steel-Critchlow-Fligner post hoc test. When comparing related samples, the Friedman test was used. Differences were considered statistically significant at *p* ≤ 0.05. For statistical analysis of the results, we used the package STATISTICA 12.0 programme modules (StatSoft, Tulsa, OK, USA) and Microsoft Office Excel 2010. After initial statistical analysis (small samples), we conducted additional studies. The stability of the results was confirmed by repeating the test on the same sample of subjects (4–5 days after the first study) with the calculation of the correlation coefficient of the product of Pearson’s coefficients. High reliability was achieved because the variance error was negligible.

## 3. Results

In this paper, we present only an analysis of the brightness temperature above the spinous processes of the lumbar vertebrae. The choice of the projection of the spinous processes is associated with their high verification during measurement. In Table 1, we show that a significant decrease in brightness temperature is noted between volunteer groups 1 and 2 (18–30 years old) and 4 and 6 (50–69 years old). Intermediate age (31–50 years) due to the large variance of the data is probably only an intermediate link. This is clear from a clinical point of view. At this moment of life, the maximum load on the spine is noted, which is also associated with possible episodes of non-specific back pain. It is likely that for an in-depth analysis of groups 3 and 4, significantly more statistics are needed.

**Figure 2:**
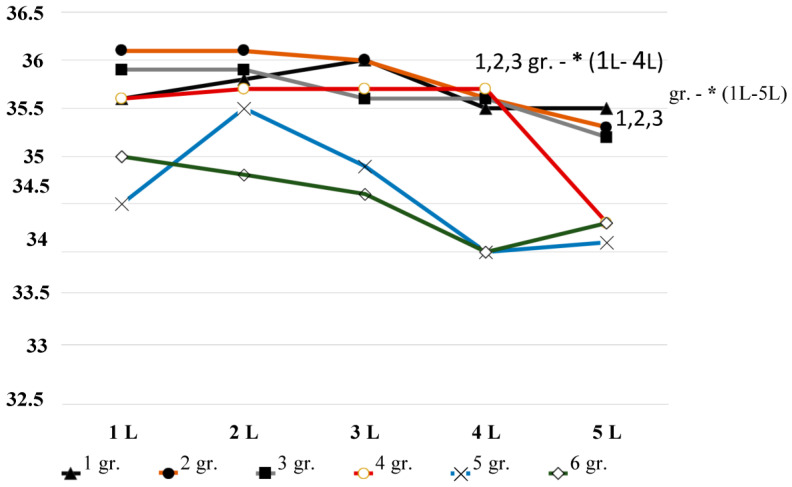
Dynamics of brightness temperature indicators above the spinous processes of lumbar vertebrae 5 in volunteers of different ages. The y-axis is temperature in °C; along the x-axis—in the first row, designation of the lumbar vertebrae; in the second row—groups. *—*p* < 0.05 between the brightness temperature of the vertebrae within their age group.

Figure 2 presents a graphical analysis of the median data (Me) of the depth temperature. The repetition of part of the table data is not random. Visual analysis allows you to see patterns that are not as visible when studying tabular data.

In age groups 1, 2, and 3 (18–40 years old), there is a significant decrease in brightness temperature between the first, fourth, and fifth lumbar vertebrae. In age groups 4, 5, and 6 (41–69 years), there is also a significant apparent trend towards lower brightness temperatures in the region of the fifth lumbar vertebra.

**Figure 3:**
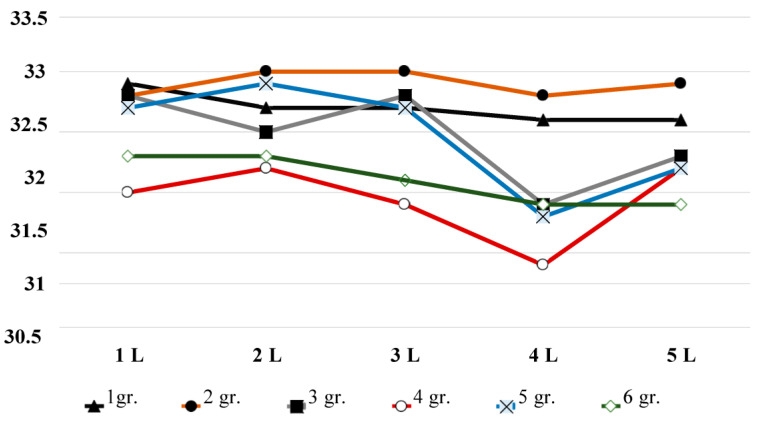
Dynamics of infrared temperature indicators over the spinous processes of the lumbar vertebrae in volunteers of various ages. The y-axis is temperature in °C; along the x-axis—in the first row designation of the lumbar vertebrae; in the second row—groups.

Analysis of infrared temperature (Table 2) and dynamics of infrared temperature indicators over the spinous processes of the lumbar vertebrae in healthy volunteers of various ages revealed (Figure 3) similar changes, once again showing the trends of brightness temperature. However, no significant changes were found. At the same time, visually, it can be seen that there is a significant trend towards a decrease in infrared temperature in areas of the fourth and fifth lumbar vertebrae, already starting at the age of 31.

A comparative analysis of the brightness temperature (Figure 2) in the first four groups shows the proximity of temperatures between the ages of 18 and 50 years, which are in the same temperature range.(Table 3) At the same time, the measurement of infrared temperature (Figure 3) set the lowest infrared temperature in group 4 (41–50 years old). In group 5 (51–60 years old), the same paradoxical situation is observed. With the expected age-related decrease in brightness temperature, which we obtained (Figure 2), when measuring infrared temperature, an increase in temperature was registered, compared with groups 1 and 2. Thus, the temperature of the infrared, in our opinion, largely depends on clothing, lifestyle, first-time back pain, etc. Age-related changes in infrared temperature in the lumbar spine, in our opinion, are not suitable to estimate biological age.

**Figure 4:**
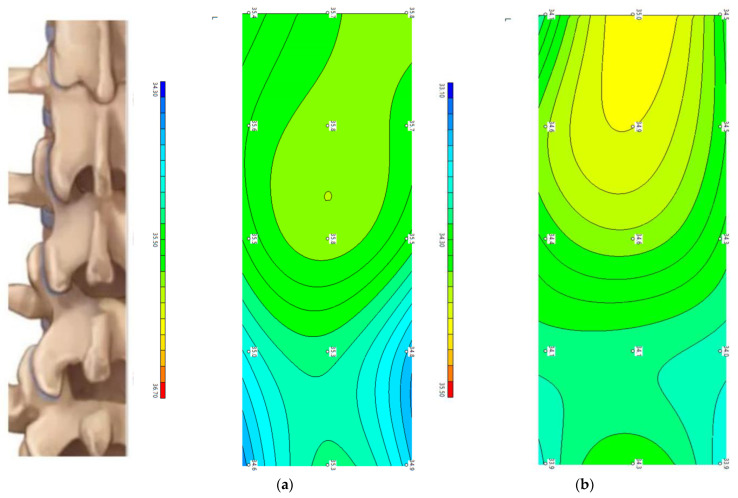
Temperature fields of brightness temperature above the lumbar vertebrae in healthy volunteers of different ages: (**a**)—group 1; (**b**)—group 6.

Figure 4 shows the temperature fields above the lumbar vertebrae in the first and sixth age groups of healthy volunteers. In general, there is a symmetrical temperature distribution and repeatability of infrared temperature with brightness temperature. This regularity disappears with any pathology [18].

**Figure 5:**
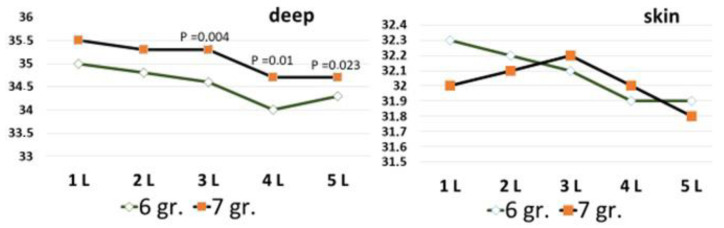
Data on the depth temperature and infrared temperature above the spinous processes of the lumbar vertebrae L1–5 in volunteers of the age groups 6 and 7: on the y axis—temperature in °C; 6 gr.—64 [63.3; 65.8] years; 7 gr.—74 [72.5; 76.5] years. The x-axis temperature in °C; along the x-axis—in the first row, designation of the lumbar vertebrae; in group 2.

Analysis of the temperature data of volunteers older than 70 requires a separate consideration (Figure 5). When comparing the indicators age groups 6 and 7, it was found that the volunteers of the older group 7 did not have the expected decrease in temperature. Even a significant increase in brightness temperature at the L3–5 level is recorded. It is very significant to observe a general increase in the minimum temperature.

There is also a statistically significant increase in the minimum temperature in the temperature measurement zones throughout the depth.

**Figure 6:**
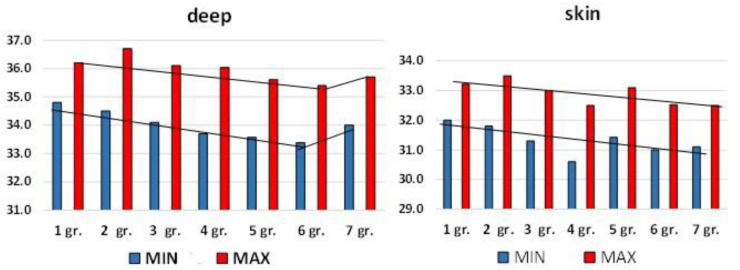
Dynamics of indicators of brightness temperature and lumbar infrared temperature vertebrae in volunteers of various ages. The y-axis is temperature in °C; along the x-axis—in the first row, designation of the lumbar vertebrae; in the second row—groups.

We analysed the indicators of brightness and infrared temperature in all age groups (Figure 6). One can clearly see the increase in the minimum and maximum brightness temperatures in group 7 compared to group 6. There is no significant difference in infrared temperatures.

## 4. Discussion

**Figure 7:**
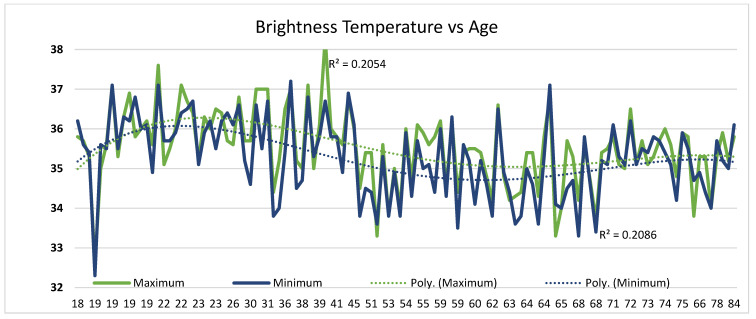
Non-linear dependence of brightness temperature (°C) on age (years).

The application of MWR has revealed a non-linear pattern of increase in brightness temperature up to 25 years old, then a gradual decrease in brightness temperature, and an increase in individuals older than 70 years old from the level of the first to the fifth vertebrae (Figure 7). The MWR method is accurate, reproducible, non-invasive, convenient, and fast. The data obtained in the present study provides a useful reference for future clinical studies of, for example, patients with non-specific low back pain (LBP), which is common and is known to become increasingly prevalent with age.

While there are general trends that are the same for all populations, individuals may age differently depending on living conditions, lifestyle, etc. There are three reasons why it could be useful to develop ageing biomarkers. Firstly, the biomarker may make it possible to predict longevity. Secondly, it may be possible to identify factors that allow timely treatment, correction, and prolongation of health [20]. Thirdly, individuals may obtain insight that allows them to function better from day to day.

The effects of ageing accumulate at the molecular and cellular level, and this can guide the search for and development of a sensitive biomarker. For example, the study of the epigenetic clock can provide information on the age of DNA methylation (DNAmAge), which may be capable of predicting all-cause mortality regardless of classical risk factors. Other potential biomarkers include the length of telomeres, repetitive DNA sequences, and closing chromosomes with a shortened time after mitosis. In addition, blood-based gene expression profiles are potential transcriptome-based predictors of age. Furthermore, proteomic predictors of age can be obtained by measuring metabolic processes such as protein glycosylation in human serum or plasma. Finally, a combination of biomarkers such as serum creatinine, glycated hemoglobin, systolic blood pressure, serum albumin, total cholesterol, serum alkaline phosphatase, and others may be the most effective way to establish biological age [21,22,23].

Up until now, there have been few studies in which measurement of infrared and brightness body temperature has been proposed as a basis for predicting the biological age of tissues. This may be because temperature measurement is being used as a proxy for measuring metabolic rate, and the relationship between metabolic rate and ageing is not well understood. Undoubtedly, changes in body temperature will affect the concentrations of poikilotherms and homeotherms. However, the relevant neuroendocrine mechanisms are complex [24], especially because thermoregulatory processes can lead to thermogenesis.

In the case of the musculoskeletal system, locomotion and the tonic and rhythmic activity of muscles can have a positive influence on biochemical processes. However, for many organs and tissues, due to their location and functions, such mechanisms do not work, and biological predictors of age should take into account possible tissue-specific differences in ageing. The lumbar spine was chosen as a focus for the present study because the region is a site of various clinical conditions, such as LBP.

The ageing of the lumbar spine is affected by two parallel but largely independent processes, namely degenerative changes in the soft tissues (e.g., muscles and intervertebral discs) and a decrease in the bone mineral density of the vertebra. Risk factors for degeneration of the intervertebral discs are age, excessive mechanical load, and genetic factors. The main changes that occur at the cellular level are increased clustering and cell proliferation. The former may reflect an attempt at repair that is thwarted by inadequate metabolite transport and interrupted by apoptosis [25,26]. There are a range of methods that may be used to study age-related changes in the vertebrae (e.g., X-ray, ultrasound, and MRI), and the main diagnostic features are radial striation, ossification, marginal bone growths (osteophytes), and osteoporosis. The synostosis of the limbus with the body of the vertebrae is completed between the ages of 20 and 30 years, and the appearance of the limbus becomes homogeneous with pronounced radial striation. By the age of 27 to 30 years, these features disappear, although osteophytes and osteoporosis are absent. Between 30 and 40 years, the contours of the vertebral bodies remain clear, but the flattening of the limbus becomes visible. Along the edges of the bodies and on the tops of the spinous processes, poorly developed osteophytes appear. After 40 years, changes may occur in the appearance of the vertebral column, including an increase in the number of osteophytes along the edges of the vertebral bodies, and by 50 years, small slit-like changes may occur in the end plates of the vertebral bodies. Between 50 and 60 years, the contours of the vertebral bodies remain relatively clear. However, the presence of osteophytes is generally pronounced, the endplates are significantly more ‘cut up’, and the height of the intervertebral discs is generally reduced. Between 60 and 70 years, the vertebral bodies start to appear much more deformed, and the limbus is interrupted to a significant extent. There is an increase in the number of osteophytes, and some degree of osteoporosis is likely to be present. As a result, the vertebrae may become somewhat transparent in appearance. By consideration of the appearance and signs described above, it is generally possible to determine the age of an individual within 5 to 7 years [27], which is like the prediction that is possible with respect to the temperature data that are presented in this manuscript.

When analysing our MWR data, there is a clear correlation between temperature changes and age-related dystrophic changes in bone tissue, especially at the age of 50 years.

Analysis of the temperature data of volunteers older than 70 requires a separate consideration.

One can clearly see the increase in the minimum and maximum brightness temperatures > 70 years old. It coincides with our results both on breasts and legs brightness temperature in healthy individuals (unpublished).

The non-linear decrease in microwave emissions with age is interpreted to have several causes, including hormonal activity, increased physical load on the spine, multi-temporal degenerative processes in the lumbar segment, osteoporosis, individual regeneration mechanisms, and genetic predisposition, whose relative importance remains to be elucidated.

When tissue is damaged, a clustering of cells frequently occurs. This clustering may represent an attempt at repair, but it is frequently disrupted by inadequate transport of metabolites and interrupted by apoptosis. Interestingly, in the intervertebral disc, populations of nucleus pulposus progenitor cells have been discovered as described by [26] and suggest that there may be opportunities for enhancing the reparative abilities of disc tissue. Another likely explanation for the age-related reduction in brightness temperature in bone tissue is a progressive decrease in bone density and a decrease in blood supply to the tissues of the bone segment.

An additional dimension to consider is that, quite commonly, while utilizing the passive technique for recording electromagnetic radiation emitted by biological tissues, a simultaneous interest often arises in investigating the impact of different forms of active electromagnetic radiation on the human body. Expanding our examination of data related to the deep temperature acquired from patients or volunteers through the MWR method, particularly in the context of the influence exerted by contemporary communication devices such as telephones, computers, and dispatch work, presents an opportunity to address a multitude of practical challenges associated with early disease prevention and treatment. This avenue of research holds great potential for addressing health concerns [28].

## 5. Conclusions

Complex behaviour is observed in the temperature of the bright tissues of the lumbar spine with increasing age. These data provide a benchmark for future clinical studies in patients with LBP and also have the potential to be used for predicting the biological age, in comparison to the chronological age, of the lumbar spine in studies of lifestyle, health, and well-being. In particular, the measurement of biomarkers, including temperature, will help in defining lifestyles that promote healthy ageing. The MWR method is accurate, reproducible, non-invasive, and fast, and it may be readily applied in studies of large cohorts.

## Figures and Tables

**Table 1:** The brightness temperature of the lumbar vertebrae above the spinous processes.

Age Group	Lumbar Vertebrae (Above the Spinous Processes), Brightness Temperatures, Average, and Minimal and Maximal Temperatures (t °C)				
	L 1 (2)	L 2 (5)	L 3 (8)	L 4 (11)	L 5 (14)
Group 1 (18–20 years old)	35.6	35.8	36.0	35.5	35.5
	[35.2; 36.1]	[35.5; 36.3]	[35.5; 36.3]	[34.8; 36.1]	[34.8; 36.0]
Group 2 (21–30 years old)	36.1	36.1	36.0	35.6	35.3
	[35.6; 36.4]	[35.7; 36.7]	[35.5; 36.3]	[34.9; 36.2]	[34.9; 35.7]
Group 3 (31–40 years old)	35.9	35.9	35.6	35.6	35.2
	[35.4; 36.9]	[35.2; 37.0]	[34.7; 36.7]	[34.4; 35.5]	[34.2; 35.5]
Group 4 (41–50 years old)	35.6	35.7	35.7	35.7	34.3
	[35.2; 35.9]	[35.4; 36.0]	[34.6; 36.0]	[33.6; 35.4]	[33.6; 35.2]
Group 5 (51–60 years old)	34.5 [34.5; 35.8]	35.5 [34.6; 35.8] (Groups 2–5) *	34.9 [34.2; 35.5] (Groups 1, 2–5) *	34.0 [33.3; 35.2] (Groups 2–5) *	34.1 [33.5; 35.0] (Groups 1, 2–5) *
Group 6 (61–69 years old)	35.0 [34.3; 35.5] (Groups 2, 3–mi6) *	34.8 [34.2; 35.4] (Groups 2–6) *	34.6 [33.8; 35.2] (Groups 1, 2–6) *	34.0 [33.4; 34.7] (Groups 1, 2–6) *	34.3 [33.8; 34.6] (Groups 1, 2–6) *

Notes: *—*p* < 0.05; numbers in brackets after the name of the lumbar vertebrae indicate the number setting of the radio antenna.

**Table 2:** The temperature of the infrared over the spinous processes of the lumbar vertebrae.

Age Group	Infrared Average and Minimal and Maximal Temperatures of the Lumbar Vertebrae (Above the Spinous Processes), t °C				
	L 1 (2)	L 2 (5)	L 3 (8)	L 4 (11)	L 5 (14)
Group 1 (18–20 years old)	32.9 [32.5; 33.5]	32.7 [32.3; 33.4]	32.7 [32.3; 33.5]	32.6 [32.0; 33.7]	32.6 [32.5; 33.8]
Group 2 (21–30 years old)	32.8 [32.3; 33.7]	33.0 [32.2; 33.5]	33.0 [31.8; 33.5]	32.8 [31.9; 33.4]	32.9 [31.6; 33.4]
Group 3 (31–40 years old)	32.8 [32.1; 33.5]	32.5 [32.0; 33.6]	32.8 [31.9; 33.3]	31.9 [31.7; 32.9]	32.3 [31.8; 33.8]
Group 4 (41–50 years old)	32.0 [31.4;32.5]	32.2 [31.0; 32.2]	31.9 [30.8; 31.9]	31.4 [30.8; 31.9]	31.3 [30.7; 33.0]
Group 5 (51–60 years old)	32.7 [31.6; 33.5]	32.9 [32.2; 33.5]	32.7 [31.9; 33.3]	31.8 [31.5; 32.8]	32.2 [31.1; 33.0]
Group 6 (61–69 years old)	32.3 [31.4; 32.7]	32.3 [31.6; 32.9]	32.1 [31.2; 32.7]	31.9 [30.7; 32.5]	31.9 [30.8; 32.6]

**Table 3:** Brightness and infrared temperature (t min and t max) of the lumbar vertebrae.

Age Group	Brightness Temperature, t °C	
	Minimum	Maximum
Group 1 (18–20 years old)	34.8 [34.4; 35.0]	36.2 [35.7; 36.8]
Group 2 (21–30 years old)	34.5 [34.1; 35.2]	36.7 [36.3; 37.0]
Group 3 (31–40 years old)	34.1 [33.1; 34.9]	36.1 [35.7; 37.1]
Group 4 (41–50 years old)	33.7 [33.2; 34.5]	36.0 [35.4; 36.5]
Group 5 (51–60 years old)	33.6 [32.9; 34.3] (Group 2–5) *	35.6 [34.7; 36.0] (Group 2–5) *
Group 6 (61–69 years old)	33.4 [32.8; 34.0] (Group 1, 2–6) *	35.4 [34.4; 35.7] (Group 1, 2–6) *
**Infrared temperature, t °C**		
Age Group	Minimum	Maximum
Group 1 (18–20 years old)	32.0 [31.4; 32.9]	33.2 [32.8; 34.0]
Group 2 (21–30 years old)	31.8 [31.1; 32.7]	33.5 [32.4; 33.8]
Group 3 (31–40 years old)	31.3 [30.7; 32.4]	33.0 [32.4; 34.3]
Group 4 (41–50 years old)	30.6 [30.2; 32.4]	32.5 [31.5; 33.3]
Group 5 (51–60 years old)	31.4 [30.4; 32.3]	33.1 [30.4; 33.5]
Group 6 (61–69 years old)	31.0 [29.9; 31.9]	32.5 [31.9; 33.2]

Notes: *—*p* < 0.05.

## Data Availability

Not applicable.
